# Mechanisms of Peptide-Induced Pore Formation in Lipid Bilayers Investigated by Oriented ^31^P Solid-State NMR Spectroscopy

**DOI:** 10.1371/journal.pone.0047745

**Published:** 2012-10-18

**Authors:** Kresten Bertelsen, Jerzy Dorosz, Sara Krogh Hansen, Niels Chr. Nielsen, Thomas Vosegaard

**Affiliations:** 1 Center for Insoluble Protein Structures (inSPIN), Interdisciplinary Nanoscience Center (iNANO), Department of Chemistry, University of Aarhus, Aarhus, Denmark; 2 Department of Engineering, School of Engineering, University of Aarhus, Aarhus, Denmark; University of Cambridge, United Kingdom

## Abstract

There is a considerable interest in understanding the function of antimicrobial peptides (AMPs), but the details of their mode of action is not fully understood. This motivates extensive efforts in determining structural and mechanistic parameters for AMP’s interaction with lipid membranes. In this study we show that oriented-sample ^31^P solid-state NMR spectroscopy can be used to probe the membrane perturbations and -disruption by AMPs. For two AMPs, alamethicin and novicidin, we observe that the majority of the lipids remain in a planar bilayer conformation but that a number of lipids are involved in the peptide anchoring. These lipids display reduced dynamics. Our study supports previous studies showing that alamethicin adopts a transmembrane arrangement without significant disturbance of the surrounding lipids, while novicidin forms toroidal pores at high concentrations leading to more extensive membrane disturbance.

## Introduction

The increasing appearance of multidrug resistant bacterial strains during past decades has caused tremendous problems with treating bacterial infections. In the past 50 years, the resistance to every new antibiotic appeared within a few years of its introduction [1] and has been responsible for increased mortality and morbidity of patients as well as for increased treatment costs [2]. This fact has prompted scientists to search for alternative and potentially more effective antimicrobial agents with novel mechanisms of action. A promising alternative includes so-called antimicrobial peptides (AMP’s) [3]. These short peptides (∼10–60 amino acid residues) [4] are abundant in Nature, constituting part of the innate immune response towards invading pathogens in a broad spectrum of organisms [5]. Among the most extensively studied AMP’s range peptides such as alamethicin [6], pardaxin [7], PGLA [8], gramicidin [9], and novicidin [10] to name a few.

The antimicrobial properties of AMP's are determined by their interactions with the lipid membranes of microorganisms [11]. Several models of such interactions have been formulated. Among them is the barrel-stave model in which a pore or an ion-channel is formed in the membrane, without significant involvement/perturbation of the lipid molecules [12], [13]. In the alternative carpet/toroidal-pore model, the peptides accumulate at the surface of the lipid membrane. For some peptides, this accumulation itself leads to cell lysis (carpet model), and for other peptides, the peptides reach a critical concentration and provoke membrane curvature, followed by formation of a toroidal pore lined with both peptide and lipids [14], [15].

The ability to characterize the interaction between AMP's and the membranes is essential to understand their mode of action and use this knowledge to modify existing or develop new peptides with other properties. Oriented sample ^15^N solid-state NMR spectroscopy is a well-established tool to study the structure/conformation of membrane-bound peptides and proteins [16], and even the peptide-menbrane dynamics may be characterized in detail [17]–[20]. The major drawback in such studies is that they target the peptide, which must be isotope labelled and abundant in quite high concentration due to the low sensitivity solid-state ^15^N NMR spectroscopy.

An alternative route to information on the AMP's modes of action is to study the perturbations of the membrane lipids upon interaction with the AMP. For this, ^2^H solid-state NMR of deuterated lipids has been extensively used to study such interactions [21]–[24]. While ^2^H solid-state NMR also requires isotope labelling, ^31^P oriented solid-state NMR seems to be the most versatile tool [25]–[27], and recently, Wi and Kim [28] presented a dynamics model for detailed interpretation oriented ^31^P solid-state NMR spectra of lipids.

In this article, we use oriented ^31^P solid-state NMR to characterize the perturbations of the lipid membranes upon addition of AMP's to establish models of their interactions with the membranes. This allows to study peptide-membrane complexes in a large range of protein:lipid (P:L) ratios. Our study uses two model AMP's: The first is the highly cationic peptide novicidin, which is a 18 residue long synthetic peptide derived from sheep cathelicidin SMAP-29 [29]. Its mode of membrane perforation is debated but commonly believed to involve a toroidal pore or carpet-like mechanism [10]. The second peptide, alamethicin, is produced by the fungus *Trichoderma viride* [30], [31] and displays antibiotic properties similarly to novicidin. This peptide presumably forms a barrel-stave type arrangement at high concentration when added to neutral and charged lipid bilayers [18], [30], [32]–[39]. The results support these two different proposed modes of action for the two peptides and reveal details of the AMP's paths to membrane disruption and pore formation through characterization of the low-P:L states.

## Materials and Methods

### Fast Rotational Diffusion of Lipids

The chemical shift observed in an NMR spectrum may in addition to its isotropic value contain information about an anisotropic part which in turn depends on the size and the orientation of the chemical shielding tensor relative to the magnetic field. This geometrical dependency may be expressed by a second-rank tensor which in the case of chemical shielding depends on the isotropic chemical shift, the chemical shift anisotropy, and the chemical shift asymmetry, as well as three Euler angles describing the tensor orientation relative to the external magnetic field. In the case of fast isotropic motion, as for small molecules in solution, the anisotropic part of the tensor is averaged out leaving only the isotropic chemical shift for direct observation. For rigid solids, the observed chemical shift entirely reflects the chemical shielding tensor. In the intermediate case of slow or non-uniform molecular reorientation, the anisotropic character of the interaction is maintained but may be reduced by motional averaging. In this case, the NMR observation will reflect a partially averaged (reduced) tensor [18], [36], [40]–[43]. Consider, for instance, phospholipid molecules in a lipid bilayer. In a hydrated environment, these molecules form lipid bilayers in which the individual molecules diffuse laterally around the bilayer normal. The lateral diffusion itself has no impact on the observed chemical shift tensor parameters, but along with the lateral diffusion comes rotational diffusion with diffusional correlation times in the µs range. The rotational diffusion averages the anisotropic chemical shift tensor to an axially symmetric tensor with reduced anisotropy and with an orientation parallel to the rotation axis, i.e. the bilayer normal. The scaled chemical shift tensor, and thereby the observed chemical shift, reports on the orientation of the individual lipid molecules in the bilayer. This fact may particularly easily be exploited experimentally using lipid bilayers oriented macroscopically with the bilayer normal parallel to the external magnetic field. Macroscopic orientation may, for example, be obtained by aligning hydrated lipid bilayers between glass plates or using bicelles which spontaneously orient with the bilayer normal perpendicular to the magnetic field, as exploited routinely in numerous solid-state NMR studies of membrane proteins [38], [44], [45].

**Figure 1:**
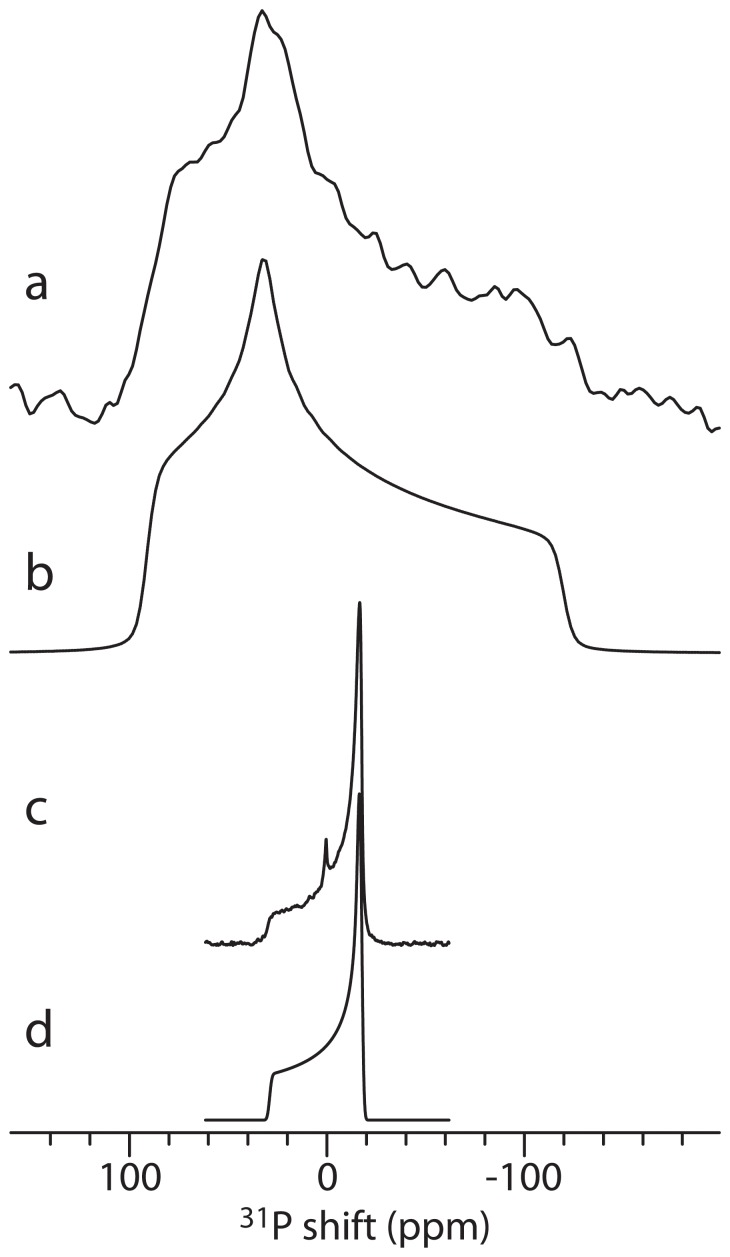
Experimental (a) and simulated (b) 31P powder NMR spectra of lyophilized DMPC. Experimental (c) and simulated (d) ^31^P static powder NMR spectra of DMPC vesicles. The peak at 0 ppm in (c) originates from the phosphate buffer.

The observation of motionally reduced chemical shift anisotropies has been extensively used to determine the membrane-bound conformation of peptides by measuring the scaled nuclear spin interaction parameters of, e.g. ^15^N and ^13^C spins belonging to peptides diffusing in lipid bilayers [36], [46], [47]. In this study, we address instead attention to the scaling of the ^31^P resonances from phosphate headgroups of lipids in presence of the peptides. Figures 1a and 1b shows experimental and simulated ^31^P powder spectra of dry, lyophilized DMPC (1,2-dimyristoyl-sn-glycero-3-phosphocholine), respectively, represented by the chemical shift parameters 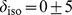
ppm, 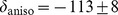
ppm, and 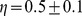 (see definition of parameters in, e.g., Ref. [48]), in agreement with previously determined parameters for similar compounds [49], [50]. In a hydrated bilayer, rotational diffusion of the lipids reduces the anisotropic chemical shift parameters to 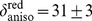
ppm with the expected axial symmetry (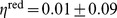) as illustrated by experimental and simulated static powder ^31^P NMR spectra of the same lipids in Figs. 1c and 1d, respectively.

### Diffusion Model

**Figure 2:**
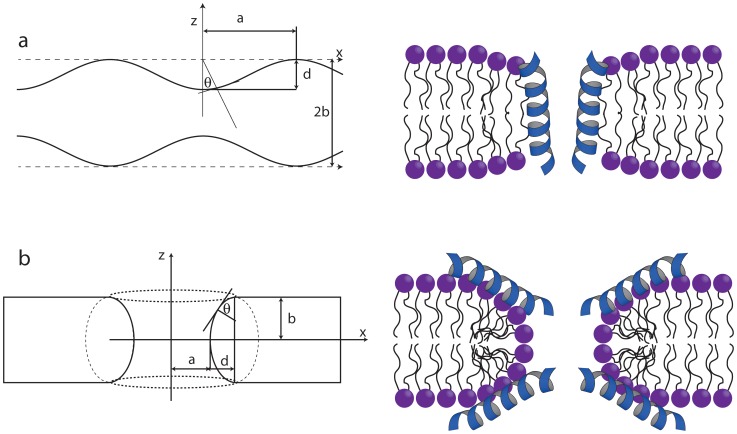
Sketches of the geometries (left column) and examples of membrane/AMP systems described by the geometries (right column). The geometries are referred to a thinned bilayer (a) and toroidal pore (b) [65] and are identical to those proposed by Wi and Kim [28]. See text for further description of the parameters.

Since antimicrobial peptides interact with lipid bilayers and induce defects which alter the geometry of the bilayer, it appears rational to supplement observations of chemical shielding parameters for specific spins (e.g., ^15^N) in the peptides with ^31^P solid-state NMR data for the lipids in which the peptides are embedded to detect the equally probable alternation (defects) of the membranes in proximity of the lipid phosphate headgroups. In this study, the perturbation of the membranes by AMPs is investigated by orienting the bilayers mechanically between glass plates with the bilayer normal parallel to the external magnetic field. Figure 2 shows models/schemes of two simple membrane geometries, which one could expect to occur in a perturbed bilayer. A thinned bilayer may be modeled as shown in Figure 2a, while a toroidal pore may be modeled by the geometry in Figure 2b. Later, we will see that the barrel-stave model, in which the peptides penetrate the bilayer without significant perturbation of the bilayer are best modeled by a thinned bilayer with a reduced diffusion rate.

To simulate the ^31^P spectra using the geometric arrangements of the lipids sketched in Fig. 2, we assume that the lipid molecules are placed at different locations on these geometries, i.e., a molecule *j* occupies a position characterized by the coordinate *x_j_* in the thinned bilayer geometry (Fig. 2a) and *θ_j_* in the toroidal pore geometry (Fig. 2b). The effects of motional averaging on the ^31^P spectrum may be described using a simple exchange matrix model reflecting the exchange between the different molecules [28]. In this model, the free-induction decay (FID) is calculated by solving the Bloch-McConnell differential equation
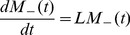
(1)where 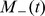
represents the magnetization vector for the ^31^P detectable single-quantum coherences of the molecules included in the model. The FID is obtained by summing the vector elements 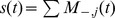. The exchange matrix is given by



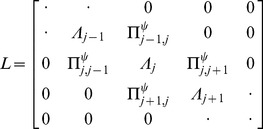
(2)In this model, the diffusion is represented by the exchange rates, 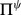. These are proportional to the latteral diffusion constant, and are quite complex functions of the angle *ψ*, that relates to the geometries as *ψ_j_* = *x_j_* for the thinned bilayer and *ψ_j_* = *θ_j_* for the toroidal pore (cf., Fig. 2). A more extensive set of expressions and associated explanations can be found in a recent paper of Wi and Kim [28]. The diagonal elements, 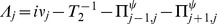, contains the frequency encoding in the first term (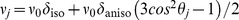) (*ν*_0_ denotes the Larmor frequency), transverse releaxation in the second term, and compensation for the off-diagonal elements in the two last terms.

The simulated NMR spectra are sensitive to the diffusion rate and the geometrical parameters in Fig. 2. In the simulations, we have assumed a bilayer thickness of 2*b* = 40 Å and have chosen a fixed value for *a* at 20 Å [28] and only optimized *d*, because the spectral changes induced by changing these three parameters are correlated. For the thinned bilayer, it is the ratio between *a* and *d* that defines the spectral appearance, while the simulations are independent on *b*. For the toroidal pore geometry, the spectral appearance depends on both the ratio between *d* and *b* and on the pore dimension *a*.

### Simulations of ^31^P Solid-state NMR Spectra

Simulations based on the above diffusion model are implemented in MATLAB [51], while the fitting of experimental spectra by interfacing the MATLAB program with the iterative fitting routines of the open-source solid-state NMR simulation program SIMPSON [48], [52], [53].

The simulated spectra contain a number of different components, e.g., thinned bilayer and toroidal pore. The relative intensities of these components are determined analytically. Assume that the simulated spectrum consists of *n* components, *S_j_*

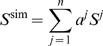
(3)where *a^j^* and *S^j^* represents the scaling (intensity) and spectrum of component *j*, respectively. The coefficients *a^j^* are chosen to yield minimal deviation between the simulated and experimental spectrum, measured by the RMS deviation
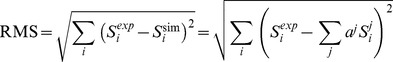
(4)where the index i refers to the i'th point in the spectrum. The coefficients yielding the minimal RMS deviation fulfill the equations
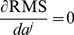
(5)for all coefficients. By differentiation, we find that this implies



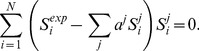
(6)The coefficients are obtained by solving the linear system, *Y* = *aX*, where
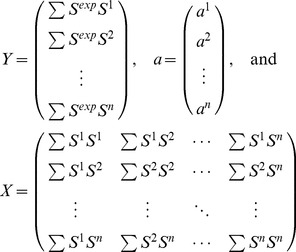
(7)


These equations are implemented as a Tcl library loaded into SIMPSON. SIMPSON and the Tcl libraries are available for download from our web site http://bionmr.chem.au.dk.

### Sample Preparation

Alamethicin (sequence in one-letter code: Ac-UPUAU AQUVU GLUPV UUEQ-Phol, Ac: acetyl, U: α-amino isobutyric acid, Phol: Phenyl alaninol) was synthesized as previously reported [54] and novicidin (sequence in one-letter code: KNLRR IIRKG IHIIK KYF) was kindly provided by Novozymes A/S (Bagsværd, Denmark). 1,2-dimyristoyl-sn-glycero-3-phosphocholine (DMPC) and 1,2-dimyristoyl-sn-glycero-3-phospho-(1'-rac-glycerol) (sodium salt) (DMPG) was purchased from Avanti lipids (Birmingham, AL). 10 mg of DMPC was used for the alamethicin samples, while 2 mg DMPG and 8 mg DMPC was used for the novicidin samples. In each case the lipids was solubilized in 156 µL MeOH together with the dry powder of the relevant peptides. The solution was pipetted onto six 8 mm × 12 mm × 0.06 mm glass slides (Marienfeld, GmbH) and exposed to air until they appeared dry. Subsequently, the glass slides were placed in vacuum overnight to ensure complete removal of residual organic solvent. Then the samples were stacked and hydrated at 37°C in an exicator containing water (100% humidity) for 48 h. Finally, the samples were wrapped in Parafilm and the NMR experiments were carried out immediately. The ^15^N NMR spectra of alamethicin and novicidin were collected from a sample with P/L = 1/15 using of 5 mg of peptides was mixed with 25 mg DMPC:DMPG in 4∶1 ratio, dissolved in MeOH and spread onto glass slides as described above.

### NMR Experiments

All solid-state NMR experiments were carried out on a Bruker-400 Avance wide-bore spectrometer (Bruker BioSpin, Rheinstetten, Germany). The ^31^P experiments were performed at 162.5 MHz in a Bruker flat-coil probe tuned in double resonance mode to ^1^H and ^31^P. An 8 µs excitation pulse was followed by 25 ms acquisition under which 40 kHz proton decoupling were applied. The repetition delay was 3 s and 512 transients were recorded for each sample. The spectra were processed using 100 Hz line broadening and referenced to a 10 mM phosphate buffer pH = 6.7 at 25°C at zero ppm. The ^15^N spectra were recorded using a ^1^H to ^15^N cross-polarization (CP) experiment with 500 µs contact time, ∼40 kHz rf field strength on both channels with a 80–100% ramp on ^1^H, followed by 25 ms acquisition with 40 kHz SPINAL-64 decoupling [55]. All experiments were carried out at 25°C.

## Results and Discussion

### Pore Formation by Alamethicin and Novicidin at High P:L Ratios

**Figure 3:**
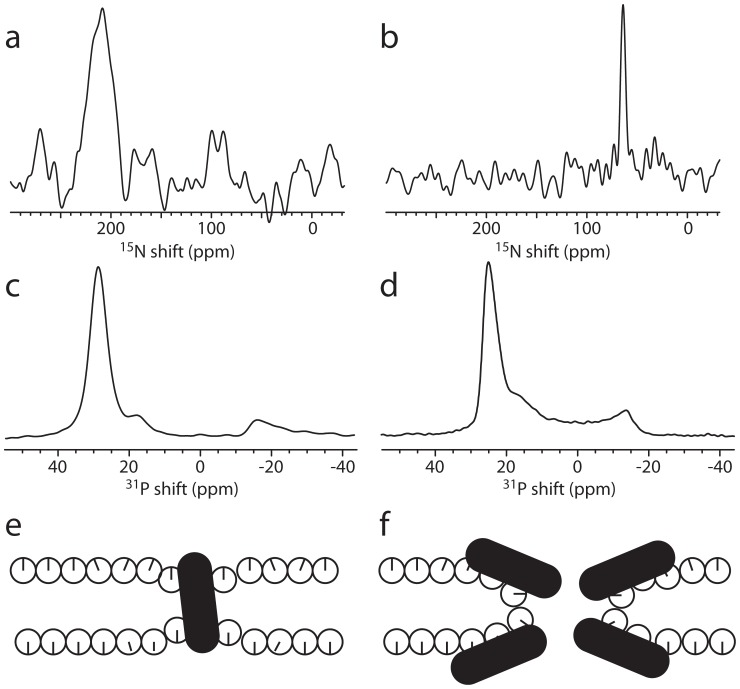
Oriented-sample solid-state ^15^N and ^31^P NMR spectra of (a) ^15^N-Aib8 labeled alamethicin incorporated into oriented DMPC lipids at a peptide:lipid molar ratio of 1∶15 and (b) ^15^N-Ile14 labeled novicidin in oriented DMPC:DMPG (molar ratio 4∶1) bilayers at 1∶15 peptide:lipid molar ratio. The resonance in the spectrum of alamethicin is substantially broadened due to mosaic spread [37] and the heterogeneous nature of the peptide-lipid interactions of the peptide [39], [56]. (c,d) ^31^P spectra of (c) a sample of alamethicin in oriented DMPC lipids with a peptide:lipid ratio of 1∶25 and (d) the novidin sample in (b). (e,f) Models showing the most likely conformations of the peptides and lipids at high peptide:lipid ratio for (e) alamethicin and (f) novicidin in lipid bilayers.

Before turning to the ^31^P investigations of the two peptide-lipid complexes, we investigated the pore formation of alamethicin and novicidin at high P:L ratios. At high P:L ratios, oriented-sample ^15^N solid-state NMR is an ideal technique to probe the membrane-anchored conformation of the peptides [16]. We prepared samples of ^15^N-Aib8 alamethicin and ^15^N-Ile14 novicidin at high peptide-to-lipid (P:L) ratios (∼1∶15) and recorded the oriented-sample ^15^N solid-state NMR spectra shown in Fig. 3a and 3b. These spectra display a clear signature of oriented samples, i.e., the spectra show narrow peaks and no powder patterns, although the resonance for alamethicin is somewhat broad due to the inhomogeneous nature of the alamethicin pores and mosaic spread [37], [39], [56].

The resonance frequency of ∼200 ppm for alamethicin (Fig. 3a) indicates a trans-membrane orientation of alamethicin at this peptide concentration, in agreement with numerous previous studies suggesting this peptide to form barrel-stave ion channels [18], [30], [32]–[39]. The ^31^P spectrum of the same sample (Fig. 3c) has the main intensity in a peak at ∼30 ppm, which corresponds to a bilayer with the bilayer normal aligned along the magnetic field and less intense peaks at 18 and -16 ppm. The two latter peaks are attributed to lipids in thinned bilayer with reduced diffusion (*vide infra*) and lipids of perpendicular orientation, respectively. These results suggest that alamethicin has a transmembrane orientation and that the lipids with reduced diffusion are involved in the anchoring of the peptide as shown in Fig. 3e. This is in agreement with the barrel-stave model, although we cannot determine the oligomerization of alamethicin from the present data.

Turning our attention to novicidin, all the intensity in the ^15^N spectrum (Fig. 3b) of the oriented sample at high P:L ratio is in a sharp peak at 64 ppm corresponding to a nearly planar orientation of the peptide, suggesting that this peptide exerts its antimicrobial action through the carpet or toroidal pore model. The distinction between these modes of action cannot be done on basis of the ^15^N spectrum alone. The ^31^P spectrum in Fig. 3d shows interesting features: (*i*) significant intensity around 25 ppm corresponding to oriented bilayers and (*ii*) significant intensity in the range −16 to +20 ppm with intense singularities at the two extrema. This does not resemble the characteristic spectra of neither an oriented sample nor a powder sample, but with the present models, it may be modeled with the toroidal pore model (*vide infra*), in agreement with other experimental data [29].

It is well established that antimicrobial peptides may introduce non-lamellar lipid phases [57], [58] or in other ways trigger membrane disruption, the latter being an essential mode of action of most antimicrobial peptides. We observe that the alamethicin samples do not change for several days, after which the humidity decreases and un-oriented and non-hydrated lipids are observed [59]. For the novicidin samples, we observe a build-up of a sharp peak at 0 ppm in the ^31^P spectrum after approximately half a day, which is attributed to non-lamellar components or other small lipid assemblies. With the present scope being to investigate the insertion of peptides, we will not address the time-evolution of the samples further, but focus on the short-term impact of the peptides at different P:L ratios.

### Alamethicin Insertion in Oriented Lipid Bilayers

**Figure 4:**
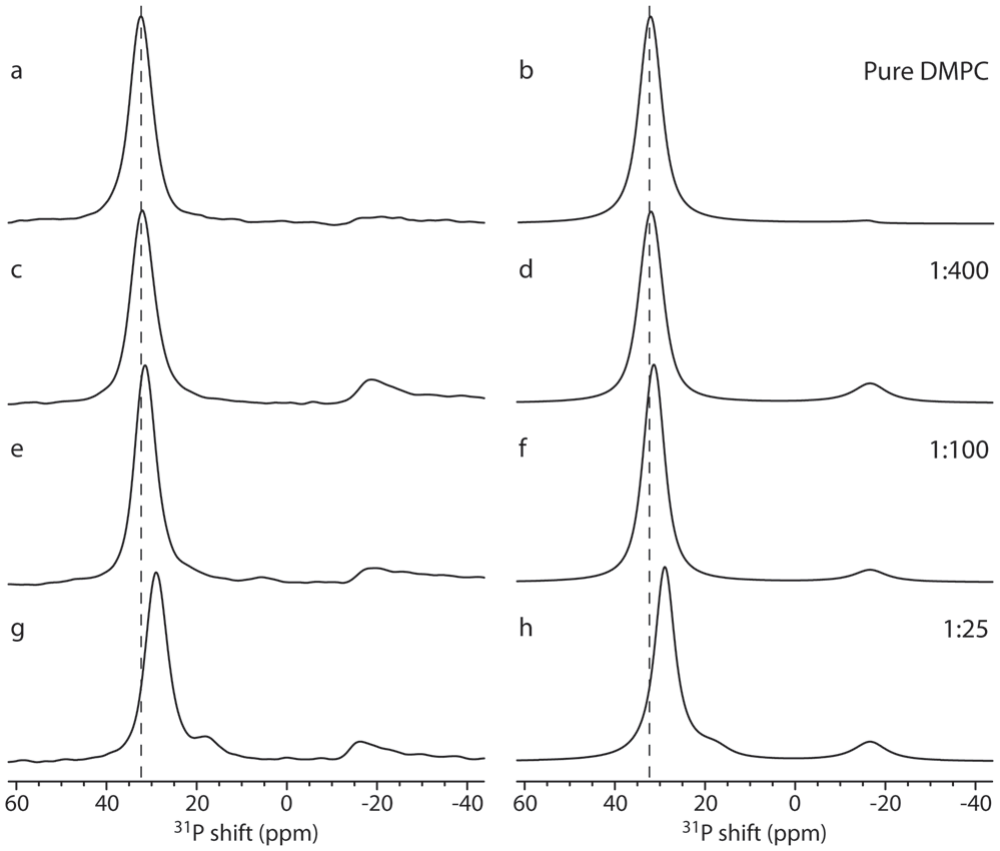
Oriented-sample solid-state ^31^P NMR spectra of DMPC lipids with increasing ratios of alamethicin relative to lipid (P:L ratios given to the right in the figure) and numerical simulations of these spectra. (a,c,e,g) Experimental spectra. (b,d,f,h) Simulated spectra. (a,b) Pure DMPC bilayers. (c,d) P:L = 1∶400. (e,f) P:L = 1∶100. (g,h) P:L = 1∶25. The spectrum at highest P:L ratio (g) is identical to Fig. 3c.

Having established reasonable models for the high-P:L peptide anchoring in consistency with previously suggested models, we will now investigate both the low-P:L and high-P:L regions by ^31^P oriented solid-state NMR to establish more insight into the membrane anchoring mechanisms of the peptides. To study these features for alamethicin, several samples containing mixtures of alamethicin and lipids with different P:L molar ratios were prepared under careful attention to the procedures leading to the reference high-P:L samples. The ^31^P solid-state NMR spectra for these oriented samples are shown in Figure 4 along with simulations employing the above diffusion model with the parameters listed in Table 1. For the different samples, only the amount of alamethicin was changed but all contained the same amount of lipids, thereby varying the P:L ratio from 1∶400 to 1∶25. The peaks at 15–30 ppm may be simulated using the thinned bilayer model, while the peaks at −16 ppm attributed to lipids with a perpendicular orientation are represented by Gaussian lines. The use of thinned bilayer geometry is rationalized by assuming that regions of the lipid bilayer distant to peptides or with surface-bound peptide can be modeled by the thinned bilayer model (Fig. 2a) with a normal diffusion rate (10^−8^ cm^2^/s) [28], [60] while regions of the lipid in closer contact with the peptides also display thinning but have reduced dynamics (10^−10^ cm^2^/s). We note that the dependence on the diffusion rate is not as pronounced as the dependence on the geometrical parameters defined in Figure 2, so the diffusion rates of 10^−8^ cm^2^/s and 10^−10^ cm^2^/s should not be taken as absolute values, but represent the order of magnitude of the diffusion rates. In addition to the spectral components discussed above, it is relevant to investigate whether the individual samples contain any unoriented components, which would be manifested as a vesicle powder pattern (cf. Figure 1c). Hence this component is included in all simulations.

**Table 1:** Parameters used for simulation of the ^31^P spectra for the alamethicin samples (Fig. 4).^a^

Sample	Geometry	Thinning^b^ (Å)	Intensity (%)	Diffusion (cm^2^/s)
DMPC	Thinning	0.57	99.9	10^−8^
	Vesicle^c^		0.1	
1∶400	Thinning	0.82	86	10^−8^
	−16 ppm^d^	–	14	–
	Vesicle^c^		0.0	
1∶100	Thinning	0.97	91	10^−8^
	−16 ppm^d^	–	9	–
	Vesicle^c^		0.0	
1∶25	Thinning	1.42	75	10^−8^
	Thinning	2.40	13	10^−10^
	−16 ppm^d^	–	12	–
	Vesicle^c^		0.0	

a All simulations assume *b = *20 Å, *a* = 20 Å (cf. Fig. 2).

b The thinning refers to the parameter *d* in Fig. 2a.

c The vesicle contribution refers to a powder pattern from a vesicle.

d This refers to the intensity at −16 ppm.

The spectrum of pure DMPC lipid sample (Fig. 4a) is characterized by a single sharp resonance at 31 ppm characteristic of oriented lipid molecules undergoing fast rotational diffusion. The peak indicates that the bilayer is oriented with the bilayer normal parallel to the magnetic field and that the bilayer is highly ordered, since the resonance approximately coincides with the leftmost edge of the vesicle spectrum (Fig. 1c). The symmetric shape of the peak indicates little or no static disorder of the bilayer [37]. Only a negligible contribution from unoriented lipids is observed. As alamethicin is added two observations are made. First and most importantly, it is observed that the intense peak at 31 ppm moves towards lower ppm values as more alamethicin is added. This highly visible effect can be modeled as an increased thinning of the bilayer as indicated in Table 1. At a P:L ratio of 1∶25, the bilayer starts to break down as observed by the appearance of slowly diffusing lipids seen in the spectra as a shoulder on the main peak. This may be explained by slowly diffusing alamethicin multimeric pore structures (e.g., barrel-stave configuration) associated with lipid molecules. This is fully in agreement with the previous observation that alamethicin is surface-bound at low concentrations and first penetrates the membrane at concentrations around 1∶25 [61]. Second, we observe the appearance of a peak at −16 ppm already at P:L ratios as low as 1∶400 (Fig. 4c). This peak appears with roughly the same intensity at all peptide concentrations and is typically observed in ^31^P spectra of lipid samples with peptides. There may be many reasons for this peak, but its characteristic resonance frequency suggests that it arises from lipid molecules oriented perpendicular to the magnetic field. We hence assume that these lipids are engaged in peptide insertion. Although un-oriented lipids would also create intensity at this frequency (see vesicle spectrum in Fig. 1c), this peak is most likely not due to un-oriented lipids, since these would create a powder spectrum with non-zero intensity in the entire region from −16 ppm to +32 ppm. More interestingly, the shapes of the resonances at −16 ppm are intriguing since they extend to lower frequencies than the expected −16 ppm extreme corresponding to the rightmost edge of the powder pattern of the hydrated vesicle sample (Fig. 1c). This tail to lower frequency suggests that the lipids no longer undergo sufficiently fast rotational diffusion around the bilayer normal to execute efficient averaging. The slow diffusion in this scenario supports our model with peptide-influenced lipid molecules aligned perpendicular to the membrane normal, since the sharp bending of the lipid bilayers increases the disorder among adjacent lipid molecules increasing the energy cost of diffusion [62]. We assume that these lipids adopt a perpendicular orientation because they are involved in the partial membrane insertion of the peptides. We address in this context attention to our previous coarse-grained molecular dynamics (MD) study of the mechanism of the alamethicin interactions with the lipid bilayer which provides a detailed discussion of how the lipid molecules help the peptides penetrate the bilayer [56]. This reveals the presence of lipids in proximity of the peptide being oriented perpendicular to the membrane normal as discussed here.

The observation of an additional slowly diffusing thinned bilayer component at high P:L ratio suggest that these lipids have reduced motional freedom because they interact strongly with the peptides. From the present data, we cannot be precise with respect to the peptide geometry. The anticipated presence of barrel-stave forming alamethicin, as previously observed in numerous studies [18], [30], [35]–[37], [63], [64], is consistent with our observations, since the barrel staves form holes in the bilayer without significant changes of the geometry, and may be regarded a limiting case of the toroidal pore model.

### Novicidin Insertion in Oriented Lipid Bilayers

**Figure 5:**
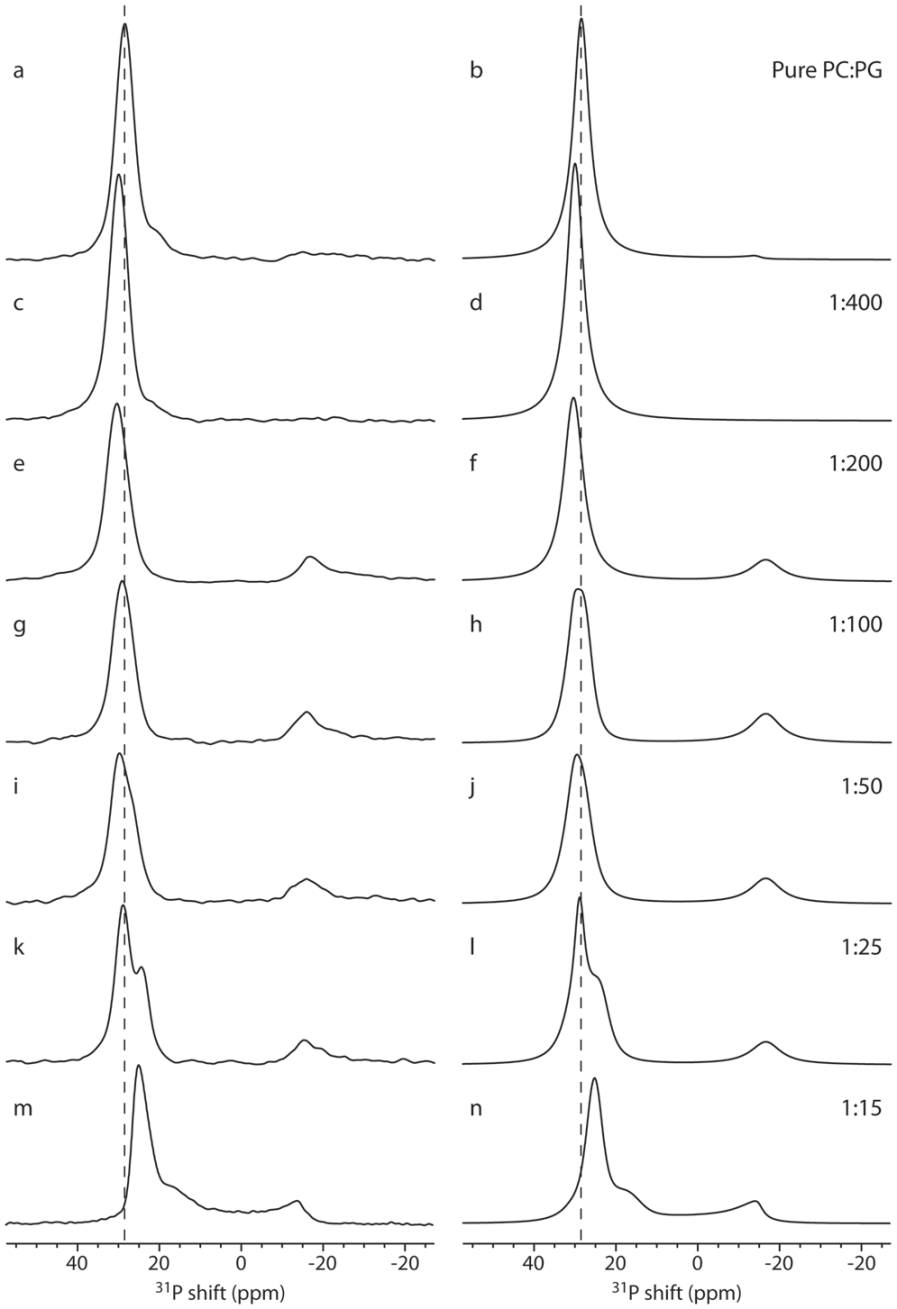
Oriented-sample solid-state ^31^P NMR spectra of DMPC:DMPG (ratio 80∶20) bilayers with increasing amounts of novicidin. (a,c,e,g,I,k,m) Experimental spectra. (b,d,f,h,j,l,n) Simulated spectra using the parameters listen in Table 2. (a,b) Pure lipid bilayers. (c,d) P:L = 1∶400. (e,f) P:L = 1∶200. (g,h) P:L = 1∶100. (i,j) P:L = 1∶50. (k,l) P:L = 1∶25. (m,n) P:L = 1∶15. The spectrum at highest P:L ratio (m) is identical to Fig. 3d.

A number of samples were prepared to study the interaction between novicidin and lipid bilayers. ^31^P oriented solid-state NMR spectra for these samples are shown in Figure 5. In all samples, the lipid bilayer consist of a mixture of DMPC:DMPG in 80∶20 molar ratio. The pure-lipid sample (Fig. 5a) displays a single sharp resonance at 28 ppm. This indicates that the lipids in the bilayer are oriented along the magnetic field axis. However, if the alignment were perfect, we would expect the peak to show up exactly at the frequency of the left edge of the powder spectrum for the DMPC vesicles (31 ppm) as observed for the pure DMPC sample (Fig. 4a). The lower resonance frequency observed here may suggests that the mixed-lipid sample has a lower order parameter of . In an alternative interpretation, we can use the thinned-bilayer model to explain the lower resonance frequency, providing a more detailed description of the system. We note that the slight disorder of the lipid is highly dynamic since the observed resonance is narrow and symmetric. Static disorder (mosaic spread) would introduce significant line broadening and an asymmetric line shape extending towards the isotropic chemical shift [37]. A fit of the spectrum (Fig. 5b) using the thinned-bilayer model shows that the peak at ∼28 ppm corresponds to lipid molecules diffusing at a rate of approximately 10^−8^ cm^2^/s in bilayers thinned by 1.5 Å as shown in Table 2. These thinning effects may be caused by the mixture of the charged and zwitterionic lipids.

**Table 2:** Parameters used for simulation of the ^31^P spectra for the novicidin samples (Fig. 5).^a^

Sample	Geometry	Thinning^b^_(Å)_	Poreshort axis (Å)	Intensity (%)	Diffusion (cm^2^/s)
PC:PG	Thinning	1.50	–	99.8	10^−8^
	Vesicle^c^			0.2	
1∶400	Thinning	1.25	–	100	10^−8^
	Vesicle^c^			0.0	
1∶200	Thinning	1.18	–	84	10^−8^
	−16 ppm^d^	–	–	16	–
	Vesicle^c^			0.0	
1∶100	Thinning	1.20	–	69	10^−8^
	Thinning	1.40	–	6	10^−10^
	−16 ppm^d^	–	–	25	–
	Vesicle^c^			0.0	
1∶50	Thinning	1.21	–	69	10^−8^
	Thinning	1.40	–	10	10^−10^
	−16 ppm^d^	–	–	21	–
	Vesicle^c^			0.0	
1∶25	Thinning	1.41	–	54	10^−8^
	Thinning	1.80	–	28	10^−10^
	−16 ppm^d^	–	–	18	–
	Vesicle^c^			0.0	
1∶15	Thinning	1.90	–	60	10^−8^
	Thinning	1.50	–	32	10^−10^
	Pore^e^	–	7.8	7	10^−12^
	Vesicle^c^			1.1	

a All simulations assume *b = *20 Å, *a* = 20 Å (cf. Fig. 2).

b The thinning refers to the parameter *d* in Fig. 2a.

c The vesicle contribution refers to a powder pattern from a vesicle.

d This refers to the intensity at −16 ppm.

e The pore short axis refers to the parameter *d* in Fig. 2b.

As novicidin is added, at a concentration equivalent to 1∶400 in molar ratio (Fig. 5c), subtle changes are observed in the ^31^P spectrum as compared to the spectrum of the pure lipids. We observe that the ^31^P shift increases suggesting a slight reduction of the bilayer thinning. This can be rationalized by the electrostatic attraction between PG lipids and novicidin, which creates a heterogeneous lipid bilayer where PC and PG lipids are no longer randomly mixed cancelling part of the mixture-induced disorder observed for the pure PC:PG sample. The spectrum can be fitted by assuming a thinned bilayer with lipids diffusing at a rate of 10^−8^ cm^2^/s in bilayers thinned by 1.25 Å as tabulated in Table 2. A doubling of the novicidin concentration to 1∶200 (Fig. 5e) induces more significant changes in the ^31^P spectrum with a further reduction of bilayer thinning, but most notable a new broad peak at −16 ppm appears. As for alamethicin, we may model this in simulations including the effect of a toroidal pore in addition to the thinned bilayer. In addition, we observe a small fraction of thinned bilayer with reduced mobility, ascribed to lipids interacting strongly with novicidin. The experimental spectrum is in good agreement with a simulation using the parameters listed in Table 2.

**Figure 6:**
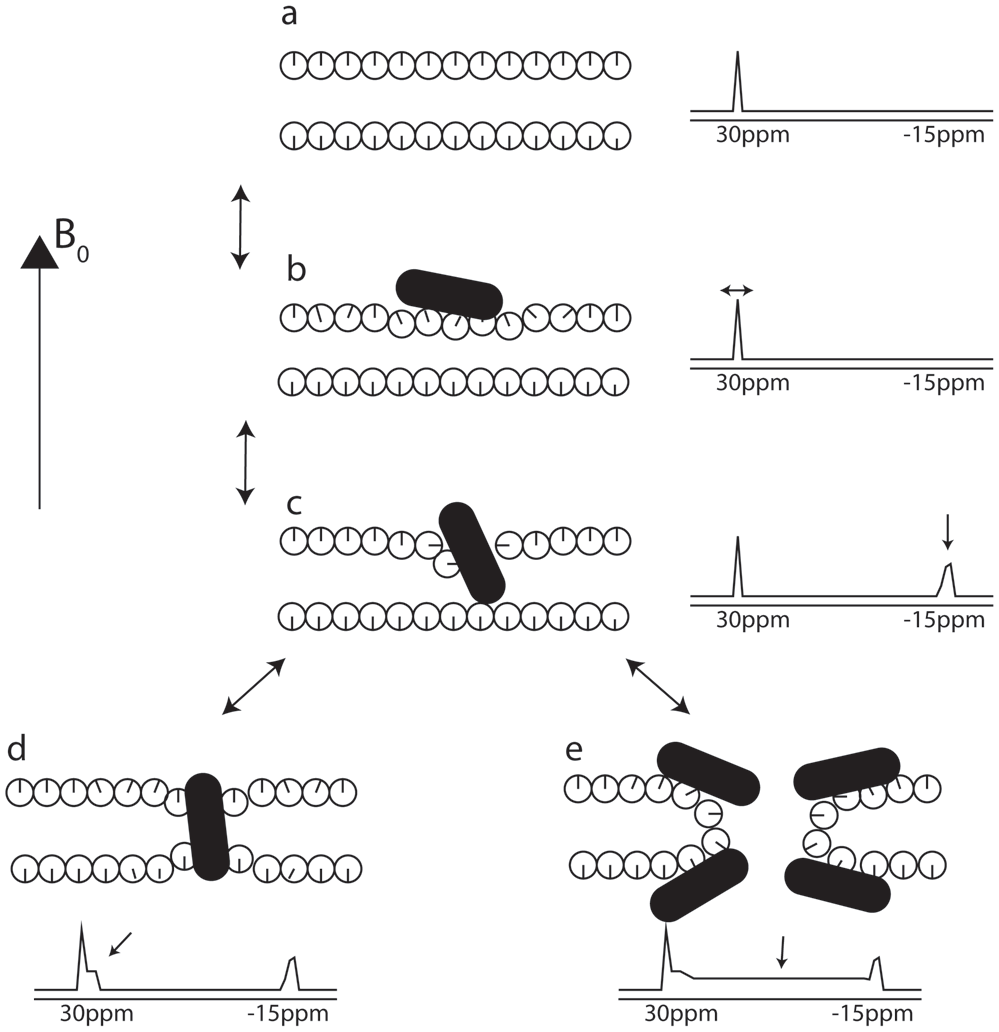
Model of peptide interactions with the lipid membrane compatible with data from oriented-sample solid-state NMR ^31^P spectra. The lipids are illustrated by circles with the radial line illustrating the orientation of the ^31^P head group. (a) Pure bilayers display a single sharp resonance at around 30 ppm. (b) At low peptide concentrations, weak interactions between peptide and lipids induce a slight disorder causing the ^31^P resonance to shift to lower values. (c) The peptide insertion involves lipids that change orientation with reduced diffusion as a consequence. This gives rise to a peak at approximately −15 ppm. (d and e) The last step in the method of action of the peptide is the penetration of the lipids and disruption of the bilayer. Alamethicin forms barrel-stave channels without significant perturbation of the lipid (d), while novicidin creates toroidal pores in the lipids (e).

At a peptide concentration of 1∶50 (Fig. 5g), the fraction of lipids participating in the peptide anchoring (the peak at −16 ppm) remains approximately at the same position and intensity, but we observe a shoulder to the ∼30 ppm peak corresponding to a slowly diffusing thinned bilayer as listed in Table 2. This component suggests a partial collapse of the lipid bilayer because of strong peptide-lipid interactions. By further increasing the P:L ratio (1∶25), this collapse is further pronounced. At the highest P:L ratio of 1∶15 (Fig. 5m), the leftmost peaks is further shifted towards lower frequency, corresponding to an increased bilayer thinning. Most notable is the appearance of intensity in the center region of the spectrum (i.e., in the region −16 ppm to 25 ppm). This observation can be modeled by the presence of a toroidal pore with a minor radius of *d* = 7.8 Å. In this spectrum, also a minor contribution from a vesicle component, corresponding to only 1.1% of the total intensity is observed. However, given the small intensity, we do not anticipate that this component plays a role in the insertion mechanism.

These results are supported by differential scanning calorimetry (DSC) measurements of DMPC:DMPG (80∶20) bilayers interacting with novicidin as reported elsewhere [29]. In the DSC study, the heat capacity of the lipid bilayer was measured with increasing amounts of novicidin present. As novicidin was added in concentrations (P:L ratios) of 1∶500 and 1∶250, the heat capacity increased which is compatible with the slight increase in thickness observed in this study at P:L of 1∶400 and 1∶200. In addition, the DSC peak broadened at P:L = 1∶250, and at approximately the same concentrations we observe a new peak at −16 ppm. At P:L = 1∶100, we observe a thickness comparable to that of the pure bilayer with an emerging new component of slowly diffusing lipids. This was also observed in the DSC study by a peak at the same position as the pure bilayer but substantially broadened. At P:L = 1∶20, the DSC measurements showed a very broad peak with a phase transition significantly lower than the pure bilayers. This is also compatible with the ^31^P spectrum recorded at P:L = 1∶15 in our study, where the spectrum is explained by a large fraction of slowly diffusing lipids in a toroidal pore geometry.

### Models for the Peptide/lipid Interaction from ^31^P Oriented Solid-state NMR

The analysis of the structure and dynamics of the lipids by ^31^P oriented solid-state NMR allows us to establish models of the peptide/lipid interactions, which in living organisms is a central feature of the AMPs. For both alamethicin and novicidin, our results may be discussed in terms of a model with an equilibrium between a series of conformations:
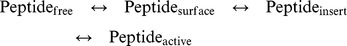



At low concentrations the peptide is free in solution (Peptide_free_). At higher concentrations, the peptide associates with the surface of the lipid bilayer (Peptide_surface_). In the case of novicidin, this can be observed with an increase in bilayer order because the highly positively charged peptide facilitates segregation of DMPC and DMPG into domains of DMPC and DMPG/novicidin, thus reducing the electrostatic repulsion between negatively charged headgroups in DMPG.

At higher peptide concentrations, a stronger interaction between the peptides and lipids are observed. Some lipid molecules change to a perpendicular orientation caused by their interactions with the peptides presumably facilitating the insertion of the peptide (Peptide_insert_). Based on the intensity of the −16 ppm peak for the two peptides, with the novicidin sample displaying the most intense peak, we deduce that the insertion mechanism of alamethicin requires less interaction with the lipids. This is supported by the fact that alamethicin is highly prone to self-aggregation (oligomer forming the ion channel) because of the hydrophobic nature of the peptide [56]. On the other hand, novicidin interacts stronger with the lipids as part of its insertion mechanism, most likely because of the strong electrostatic potential between the positively charged peptide and the negatively charged DMPG lipids.

As the concentration of peptide becomes exceedingly high, the peptides are reaching their active cell-lysing state (Peptide_active_). For alamethicin, this creates a portion of slowly diffusing lipids in a thinned bilayer. The thinning is most likely caused by the hydrophobic mismatch of the short peptide, while the hydrophobic interactions between the transmembrane peptide and lipid acyl chains reduce the diffusion compared to the free lipid bilayer. For novicidin, the growing fraction of the slowly diffusing lipids in a thinned bilayer is consistent with large domains of peptides interacting mainly with DMPG lipids due to the high positive charge of the peptide. The formation of toroidal pores at high P:L ratios is evidenced by the presence of intensity in the central part of the ^31^P spectrum and the conclusion from the ^15^N spectrum that novicidin has a conformation close to an in-plane arrangement.

Figure 6 summarizes this interpretation of our observations in a sketch of the proposed mechanism of action of the two peptides. The model involves a weak interaction between the peptide and the surface of the bilayer (Fig. 6b) causing the 30-ppm resonance to shift since the peptide perturbs the lipids. This perturbation is convincingly modeled by a thinning of the bilayer. A stronger interaction with the lipids assists the insertion of the peptide. Here, part of the lipids adopts a perpendicular orientation with a highly reduced diffusion rate as a natural consequence (Fig. 6c). In this study, we have used the toroidal pore geometry with slowly diffusing lipids to model the ^31^P spectra for this scenario. At very high peptide concentrations, we observe different modes of action for the two peptides. Alamethicin adopts transmembrane conformations (Fig. 6d) and novicidin uses the lipids to form toroidal pores with the lipid (Fig. 6e).

In the present study, the models of interaction between the peptides and lipids are established from the ^31^P oriented solid-state NMR spectra supported by prior knowledge on the high-P:L molar ratio structures obtained by alternative methods. The next step is to be able to develop such models based exclusively on the ^31^P spectra, since this would allow studies of peptides not amenable to isotope labelling. Having established these tools, a long-term goal is to be able to study such peptides in complex lipid mixtures, more closely resembling the native lipids. The present investigation of lipids with mixed PC and PG head groups represents a first step along these lines, revealing interesting characteristics of such lipids.

### Conclusion

We have used ^31^P oriented solid-state NMR experiments to investigate the interaction between antimicrobial peptides (AMPs) and lipids to establish models for their modes of action. Alamethicin and novicidin were chosen as two different AMP candidates. The two peptides differ mainly in their electrostatic charge and they are both believed to form α-helixes in lipid bilayers [29], [39]. We find that the peptides induced defects in the lipid bilayer that may be described as thinned bilayers and toroidal pores geometries. Lipids that are not affected by significantly by the peptides diffuse fast (∼10^−8^ cm^2^/s). At high peptide concentrations, alamethicin adopts a transmembrane conformation and forms channels without significant engagement/perturbation of the lipids, however with a small fraction of the lipids forming a thinned, slower diffusing bilayer. This fraction is most likely responsible for the hydrophobic anchoring of the peptide. Novicidin, in turn, forms toroidal pores at high peptide concentrations, with significant perturbation of the lipid bilayer as a consequence. Based on these results, we anticipate that ^31^P oriented solid-state NMR will find immediate applications for the study of peptide/lipid interactions of peptides that cannot be isotope labelled.
